# Metagenomic analysis reveals houseflies as indicators for monitoring environmental antibiotic resistance genes

**DOI:** 10.1111/1758-2229.70032

**Published:** 2024-11-19

**Authors:** Yuhan Yang, Ping Xu, Wei He, Fei Tao

**Affiliations:** ^1^ State Key Laboratory of Microbial Metabolism and School of Life Sciences & Biotechnology Shanghai Jiao Tong University Shanghai People's Republic of China; ^2^ Department of Respiratory Medicine, The Fifth People's Hospital of Shanghai Fudan University Shanghai People's Republic of China

## Abstract

Given the threat to public health posed by antibiotic resistance transmission, environmental monitoring is essential for tracking antibiotic resistance genes (ARGs). Houseflies, being ubiquitous organisms capable of carrying and disseminating ARGs, serve as suitable indicators for environmental monitoring. In this study, we employ metagenomic approaches to investigate housefly body surface samples from five typical sites associated with human activities. The investigation reveals microbiome diversity among the samples, along with variations in the occurrence and mobility potential of ARGs. Metagenomic analysis indicates that the composition of ARGs on housefly body surfaces is influenced by environmental ARGs, which may be enriched on the housefly body surface. The resistance genes related to multidrug, *β*‐lactam, bacitracin, and tetracycline were the predominant ARGs detected, with multidrug‐related ARGs consistently exhibiting dominance. Furthermore, the abundance of ARGs in the different housefly body surface samples was found to correlate with the population density and mobility of the sampling site. Natural environments exhibited the lowest ARG abundance, while areas with higher population density and limited population mobility displayed higher ARG abundance. This study emphasizes the effectiveness of houseflies as monitors for environmental ARGs and underscores their potential for assessing and controlling antibiotic resistance risks in urban environments.

## Graphical abstract

Housefly body‐surface samples were collected from five distinct sites associated with human activities and subjected to metagenomic sequencing analysis. The results revealed that the composition of species and ARGs on the housefly body surface reflected the characteristics of their respective environments. Additionally, the samples from different locations indicated varying levels of environmental risk.

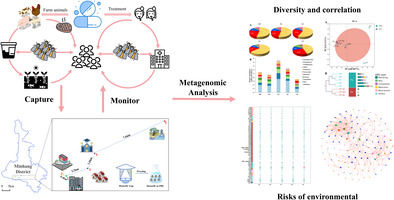

## INTRODUCTION

Antibiotics have been widely used in human treatment since their discovery in 1929. However, the excessive and improper use of antibiotics in clinical practice has resulted in the enrichment and transmission of antibiotic‐resistance genes (ARGs) in various environments. These environments encompass river estuaries, wastewater treatment plants, ponds, airborne particles, livestock farms, and even extreme environments (Dang et al., 2020; Guo et al., 2020; Gwenzi et al., 2022; Yuan et al., 2019). The widespread presence of ARGs within various environments has garnered significant global attention. ARGs can integrate into environmental microorganisms through mobile elements such as integrons and plasmids, enabling them to acquire antibiotic resistance. Furthermore, within bacteria, the transmission of ARGs occurs through horizontal gene transfer, which significantly amplifies the prevalence and risk of ARGs. Recent reports from the WHO indicate a disturbing trend of increasing drug resistance in common bacterial infections. Notably, drug‐resistant strains of *Escherichia coli*, *Salmonella*, and *Neisseria gonorrhoeae* caused a minimum of 15% more infections compared to 2017 (WHO, 2022). This data underscores the potential threat that both ARGs and their host microorganisms to human health. Consequently, it becomes paramount to diligently indicate the profiles of ARGs and their hosts in anthropogenic environments to effectively safeguard human health.

Previous studies have utilized multi‐source and multi‐point sampling to comprehensively assess environmental risks. For example, in a study examining ARGs and their hosts in a pig farm, researchers collected 27 samples from faeces, wastewater, and soil at five different sites (Zhang, Liu, et al., 2021). Similarly, scientists evaluating ARGs and potential health risks in the Danjiangkou Reservoir analyzed 24 water and 18 sediment samples from 12 sites across different seasons (Dang et al., 2020). These studies highlight the ability of multi‐source and multi‐point sampling approaches to meet research requirements. However, the results depend on the density and number of samples collected. While these approaches can be effective, they may not be as convenient as using a smaller number of samples from a single source. Nonetheless, relying on a commonly single sample is insufficient for fully assessing the overall risk profile of the environment. Therefore, an easily accessible sample that can comprehensively reflect the environmental risks is needed.

The housefly has been a part of human life for a long time (Sanchez‐Arroyo & Capinera, 2017). It is a highly reproductive insect that can be found everywhere in the environment and it is well adapted to living in human habitations (Iqbal et al., 2014). Houseflies are often abundant in areas where human activities take place, such as markets, hospitals, and restaurants (Awache & Farouk, 2016). They can carry a variety of antimicrobial‐resistant bacteria (ARB) and pathogenic microorganisms that can cause more than 50 diseases (Boiocchi et al., 2019; Fukuda et al., 2016; Khamesipour et al., 2018; Tsagaan & Okado, 2015). A study conducted in different environments of a small southern US town showed significant differences in the colonization rate of ARBs among different sampling sites (Poudel et al., 2019). Similarly, research from Belgium has shown that while the internal bacterial community of houseflies from different sampling locations exhibits relatively similar characteristics, the external bacterial communities are influenced by the specific habitat of the houseflies (Park et al., 2019). Additionally, the body surface of houseflies in hospital environments can carry important pathogens from that hospital (Kassiri et al., 2015). Previous studies have also demonstrated that houseflies in different environments can carry various ARGs on their body surface, and the ARGs they carry may be associated with their respective environments. For instance, houseflies can contribute to the maintenance of antibiotic resistance in farms (Fukuda et al., 2019). Based on their close relationship with humans and their ability to carry ARGs and pathogens, houseflies have the potential to serve as a comprehensive and convenient environmental risk indicator.

Metagenomic analysis is a powerful method that allows for obtaining comprehensive information about microbial communities through the assembly of genomes and prediction of functional attributes from metagenomic sequencing results. Moreover, metagenomic analysis enables the assessment of the risk and transmission of ARGs by comparing sequencing results with known databases of ARGs or mobile genetic elements (MGEs). In previous studies, metagenomic sequencing has been instrumental in examining risk profiles across various environments and samples, offering valuable insights. For instance, it has shed light on the changes in the association of ARGs with MGEs in wastewater and activated sludge (Dai et al., 2022). Additionally, metagenomic analyses have enabled the exploration of the abundance, diversity, and distribution of ARGs and their potential bacterial hosts (Su et al., 2022). Therefore, metagenomic might be suitable for exploring the potential of the houseflies to serve as an environmental indicator.

In this study, we collected houseflies from five typical sites associated with human activities, to investigate their potential as environmental indicators. Metagenomic sequencing analysis was conducted on the housefly body‐surface samples to explore the species composition and characteristics of ARGs. The associations and risk profiles of ARGs found on the housefly body surfaces were then analyzed alongside those detected in environmental samples. Furthermore, we characterized the species that carry ARGs on the body surface of houseflies and investigated the coexistence of MGEs and ARGs in the samples. These results can help assess the suitability of houseflies as environmental monitors and contribute to the improvement of public health surveillance and risk assessment practices.

## EXPERIMENTAL PROCEDURES

### Site description and houseflies sampling

Given that the activity radius of houseflies is approximately 328–1640 feet, we set up five sampling sites with a minimum spacing of 400 m (Stafford, 2008). Sampling was conducted at various locations associated with human activities in the administrative district, including a general hospital (with over 750 beds and 2.1 million annual outpatients, HP), Bijiang Plaza (located 0.4 km from the hospital and characterized by shopping malls, major communication routes, high vehicular traffic, and population mobility, PL), an urban community (situated 2.3 km east of the hospital with a dense population, 740 households, UC), a school (located 1.6 km northeast of the urban community, SC), and a country park (situated 7.6 km northeast of the school with low human activity, CP) (Figure S1). Housefly captures were performed using hanging housefly traps (Beijing Zhanying Technology Co., Ltd.) equipped with transparent plastic flexible boards (~24 cm × 32 cm) to prevent rain infiltration. Five different sampling sites were established in Shanghai from August to September 2020, with three housefly traps deployed at each site. The traps suspended approximately 1.2 m above the ground, were positioned in diverse areas within each sampling site, such as hospital dormitories, gardens, commercial plaza parking lots, residential waste bins, student dormitories, and park toilets. After approximately 2 weeks, housefly traps were retrieved once a minimum of 30 houseflies were collected in each trap. The traps were folded, placed in plastic bags, tightly sealed, and transported to the laboratory. Water, soil, and sediment samples were also taken from the CP site. A 1 L water sample was obtained from the Huangpu River using a plexiglass water sampler (Suzhou Ariel Plexiglass Co.). Soil samples were collected at three locations using the five‐point sampling method and then mixed well. The silt was collected at various locations using a stratified sampler and pooled into a single sample. The relevant sample information has been listed in Table S1.

The water samples were centrifuged at 4°C and 10,000 rpm, the supernatant was retained, and the precipitate was collected into 15 mL centrifuge tubes. After evenly mixing the housefly samples from the three traps at each sampling site, houseflies of similar quality were selected using sterile tweezers and transferred to a shake flask containing 100 mL of sterile PBS buffer. The shake flasks were then incubated in a shaker, which was set at 30°C and rotated at 200 rpm. The samples were later suction‐filtered using aqueous filter membranes, and the membranes with the captured samples were placed in 15 mL centrifuge tubes, rapidly frozen using liquid nitrogen, and stored at −80°C.

### DNA extraction and sequencing

Genomic DNA was extracted from 5 housefly body‐surface samples using the FastDNA Spin Kit (MP Biomedicals, Germany) according to the manufacturer's instructions. The concentration and purity of the extracted DNA were assessed using the TBS‐380 and NanoDrop2000 (Thermo Fisher Scientific, Wilmington, DE, USA), respectively. The quality of the DNA extracts was confirmed by electrophoresis on a 1% agarose gel. To achieve an average fragment size of approximately 400 bp, the DBA extracts were fragmented using the Covaris M220 (Gene Company Limited, China) for subsequent paired‐end library construction. Paired‐end libraries were constructed using the NEXTFLEX Rapid DNA‐Seq kit (Bioo Scientific, Austin, TX, USA). Adapters containing full sequencing primer hybridization sites were ligated to the blunt ends of the DNA fragments. Paired‐end sequencing was performed on the Illumina NovaSeq 6000 (Illumina Inc., San Diego, CA, USA) at Majorbio Bio‐Pharm Technology Co., Ltd. (Shanghai, China). The sequencing was carried out using NovaSeq 6000 Reagent Kits as per the manufacturer's instructions (www.illumina.com). On average, approximately 10 Gb of metagenomic data was generated per sample. Detailed information on the datasets of these samples can be found in Table S1. The generated data have been deposited into the NCBI Sequence Read Archive database under the accession number (NCBI) [PRJNA1052376].

### Raw sequencing data processing and taxonomy profiling

Paired‐end Illumina reads were subjected to adaptor trimming and the removal of low‐quality reads (length <50 bp or with a quality value <20 or with N bases) using fastp (v0.20.0) (Chen et al., 2018). The housefly genome (https://www.ncbi.nlm.nih.gov/genome/14461) was used as a reference for read alignment, which was performed using BWA (v0.7.9) (Li & Durbin, 2009). Hits associated with the reads and their mates were subsequently removed.

After quality control, the clean reads were assembled using SPAdes (Prjibelski et al., 2020), and the assembled contigs with longer than 500 bp were reserved (spades.py ‐‐meta‐t 48‐k 21,33,55,77,99,127‐1 R1.fastq‐2 R2.fastq‐o out). The quality of the assembly was assessed using QUAST (v5.0.2) (Gurevich et al., 2013). Contigs longer than 500 bp were selected for open reading frame (ORF) prediction using Prodigal (Hyatt et al., 2012). Redundancy was eliminated, and a unique initial gene catalogue was obtained using CD‐HIT (v4.7) (Li & Godzik, 2006), employing clustering at 95% identity and 90% coverage parameters (cd‐hit‐I sample.fasta‐o sample.fasta‐c 0.95‐M 640000‐T 24‐n 5‐d 0‐aS 0.9‐g 1‐sc 1‐sf 1) (Table S2).

For taxonomic analysis, clean reads were aligned to standard and fungi databases from NCBI using the Kraken2 (Wood et al., 2019). The results were summarized and relative quantification of each taxon was obtained using Bracken (Lu et al., 2017).

### ARG read mapping and identification of ARG‐carrying contigs

Metagenomic sequencing data were analyzed using the ARGs‐OAP pipeline 2.3 (Yin et al., 2018) to identify and classify ARG‐like reads into types and subtypes. In brief, ARG reads were matched against the SARG 2.0 database using a cutoff of 10^−7^ *E*‐value, 80% identity, and 75% alignment length. The ARG profiles were compared across samples at the type, subtype, and gene levels. To compare the ARG abundance between different samples, ARG abundance was normalized to 16S rRNA copies and presented as ARG copies per 16S (referred to as ‘copies/16S rRNA’). The calculation formula was as follows:

(1) $$\text{Abundance}=\sum_{1}^{n}\frac{N_{\mathrm{ARG}-\text{likesequence}}\times 150/L_{\mathrm{ARG}-\text{referencesequence}}}{N_{16S\;\text{sequence}}\times 150/L_{16S\;\text{sequence}}}$$

In Equation (1), nrepresents the total number of the ARG reference sequences mapped to an ARG type or subtype, $N_{\mathrm{ARG}-\text{likesequence}}$ is the number of ARG‐like sequences that are annotated as a specific ARG reference sequence, and $N_{16S\;\text{sequence}}$ is the number of the 16S sequences identified from metagenomic data. The length of clean reads is 150, and $L_{\mathrm{ARG}-\text{referencesequence}}$ is the length of the ARG reference sequence. The average length of the 16S sequence, $L_{16S\;\text{sequence}}$, used in Equation (1) was 1432 bp. This value was derived from the Greengenes database, which served as the reference for identifying 16S rRNA sequences using a local BLAST approach (Li et al., 2015). The relevant information on ARG types and subtypes in the present study is shown in Tables S3−S4.

Predicted ORFs were aligned to the SARG databases with ≥80% identity, *E*‐value ≤10^−5^, and ≥70% coverage to identify ARG‐like ORFs. To compare coverage between different samples, the coverage of ARG‐like ORFs coverage was normalized by the data size of each sample (copies/Gb). The calculation formula was as follows:

(2) $$\text{Coverage}=\sum_{1}^{n}\frac{N\times 150/L}{G}$$

In Equation (2), n represents the total number of ARG‐like ORFs in one sample, N refers to the number of clean reads mapped to ARG‐like ORFs. 150 is the length of clean reads, L represents the length of the target ARG‐like ORFs, and G represents the data size (Gb) of clean reads per sample (Dang et al., 2020).

The ORFs on ARG‐carrying contigs (ACCs) were annotated against the NCBI nonredundant (NR) protein database (retrieved on March 2023) using BLASTP (2.9.0) with an *E*‐value ≤10^−5^. The results were parsed with MEGAN (v6) (Huson et al., 2016), and the contigs were annotated to a specific taxon if more than 50% of the ORFs on a contig were attributed to the same taxon, using a customized Python program for voting.

Based on the results of NR annotation, MGEs located on ACCs were annotated if they matched one of the following keywords: plasmid, conjugative, integrase, integron, transposase, transposon, recombinase, recombination, conjugal, mobilization and resolvase (Forsberg et al., 2014; Shi et al., 2023). MobileOG‐db (v1.6) is a manual collection of MGEs database that has been to enhance the annotation and classification of MGEs found in contigs carrying ARGs, based on their molecular mechanisms associated with MGEs (Brown et al., 2022). Plasflow (v1.1) was used to identify the genetic location of ACCs longer than 1000 bp that are located on a plasmid or chromosome (Krawczyk et al., 2018).

Different public health risks can be associated with diverse ARGs, and these risks can be assessed based on factors such as human accessibility, host pathogenicity, and mobility. In this study, the researchers classified ARGs into four ranks: Rank I includes ARGs that are mobile and found in pathogens. Those ARGs that are genetically mobile and prevalent in human environments are designated as Rank II, and they are considered a ‘future threat’ to human health due to their potential for spread. Rank III is assigned to ARGs that, while prevalent in human‐related environments, have limited gene mobility. Finally, ARGs that do not meet any of the above criteria are categorized as Rank IV, indicating they pose the least threat to human health. (Zhang, Gaston, et al., 2021).

### Network and statistical analysis

Network analysis has been widely employed to explore potential correlations between microbial groups and genes, as well as between genes within complex microbial communities. Spearman's correlation coefficients between ARGs and microbial communities were calculated by the ‘Hmisc’ package in R and visualized by Gephi (v0.10.1). The clustering coefficient, shortest average path length, and modularity of the correlation network are further calculated in Gephi. A significance level of *p* <0.05 was used for all statistical tests. Partial results were visualized using R packages such as ‘tidyverse’, ‘ggplot2’, ‘ggpubr’, ‘patchwork’, ‘ggsci’, and ‘ggthemes’.

## RESULTS

### Species diversity in different housefly body‐surface samples

Rank abundance curves were generated to illustrate the normalized diversity of housefly body‐surface samples at the genus level (Figure S2). The results indicated variations in species richness across the five sites. Alpha diversity analyses further demonstrated differences in community richness and diversity among the samples (Figure S3). The upset plot analysis revealed that 147 species were shared among all sites, while the number of unique species ranged from 143 to 305 (Figure S4). This highlights the significant role of local ecological factors in shaping the microbial communities of houseflies. It suggests that while a basic microbiome exists, environmental variations prompt distinct microbial adaptations and diversifications on the housefly body surface.

**FIGURE 1:**
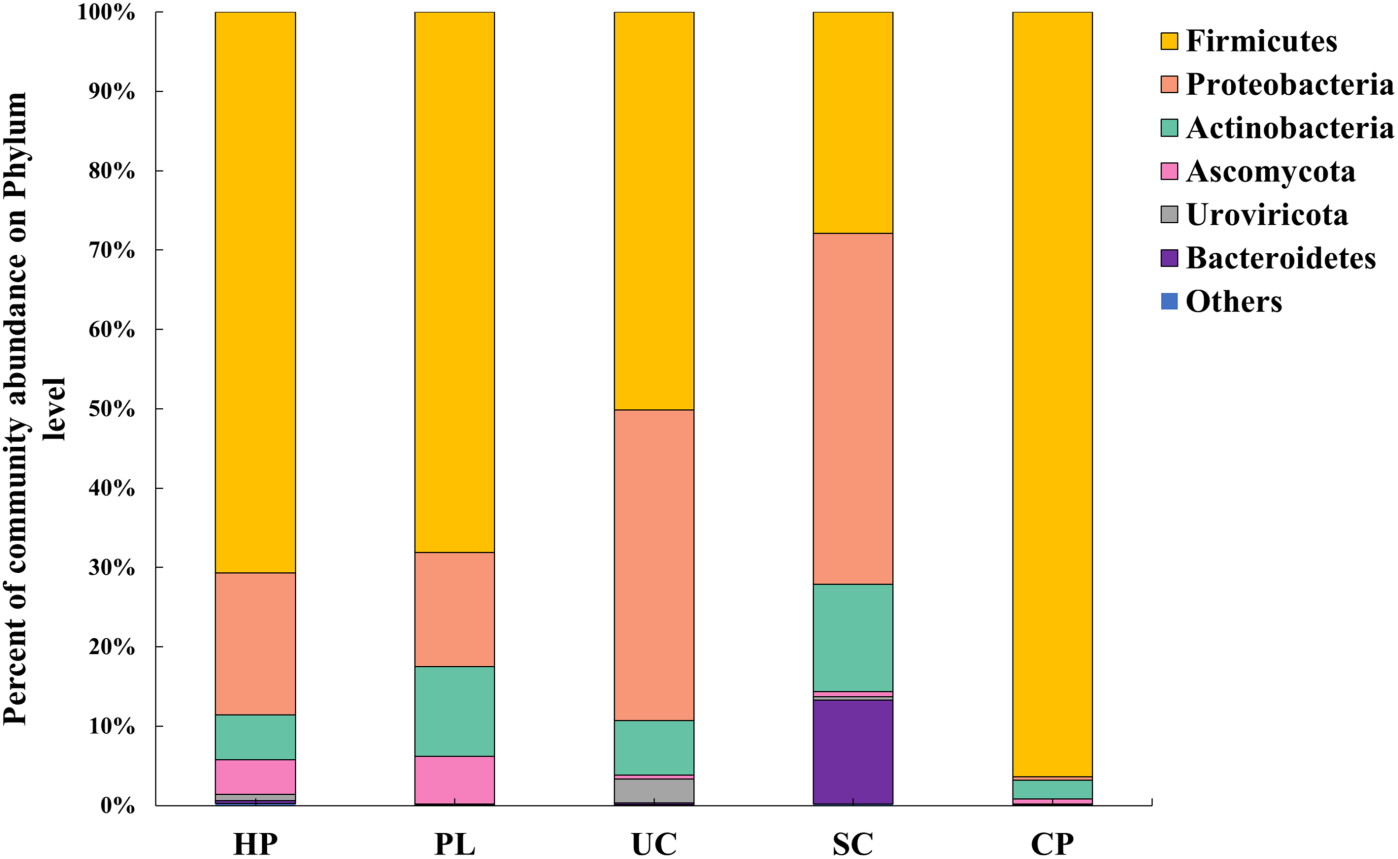
The relative abundances of bacterial phylum in the housefly body‐surface samples collected from five sites. Composition of microorganisms in the housefly body‐surface samples at phylum level. Top 6 bacteria at the phylum level in relative abundance by metagenomic analysis. Each colour represents the relative abundance of bacterial taxa on the stacked bar graph.

The bacterial community compositions at the phylum level were determined through Kraken2 annotation, as shown in Figure 1. The predominant phyla in the HP sample were *Firmicutes* (70.7%), *Proteobacteria* (17.9%), and *Actinobacteria* (5.7%). In the PL sample and the UC sample, the predominant phyla were *Firmicutes* (68.2% and 50.1%, respectively) and *Proteobacteria* (14.3% and 39.2%, respectively). The SC sample exhibited a different pattern with Proteobacteria (44.2%), *Firmicutes* (27.9%), *Actinobacteria* (13.5%), and *Bacteroidetes* (13.0%) as the predominant phyla. Notably, the CP sample displayed a distinct bacterial composition, with *Firmicutes* (96.4%) as the predominant phylum.

These results, referring to the analysis of species composition, were further supported by the PCoA plot based on the Bray–Curtis distances at the genus level (Figure S5). The plot indicated that the proximity of the HP sample and the PL sample, while the remaining samples exhibited clear separation This observation suggested significant variability in the species composition structure among the samples.

**FIGURE 2:**
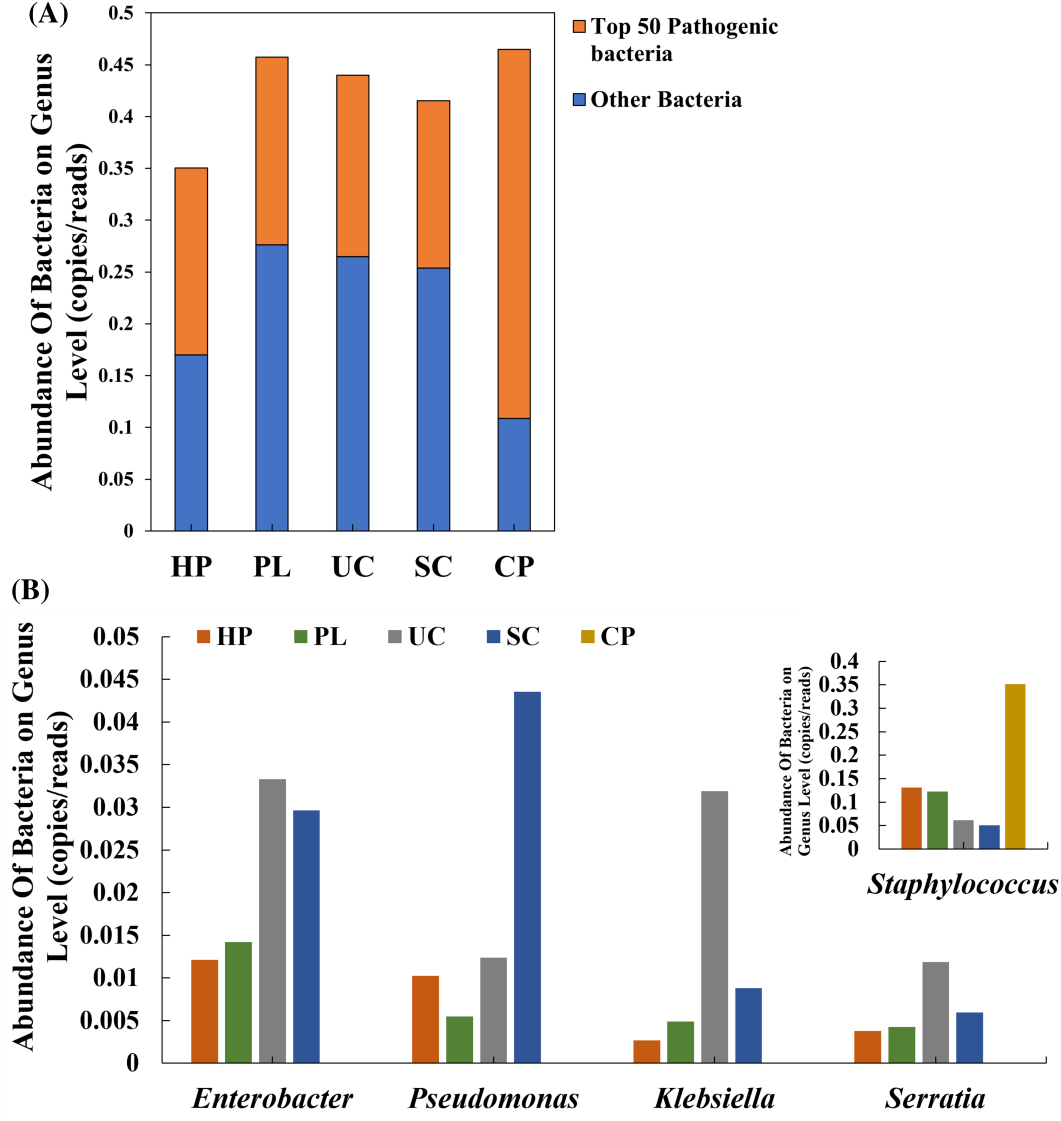
Abundance of pathogenic bacteria at the genus level in the housefly body‐surface samples collected from five sites. (A) Abundance of top 50 pathogenic bacteria and other microorganisms. (B) Abundance of top 5 pathogenic bacteria. *Staphylococcus* was plotted separately due to excessive variation.

The Kraken2 annotation at the genus level provided normalized results based on clean reads, which were then sorted by abundance to identify the top 50 pathogens among them (Table S3). The abundance results (Figure 2A) revealed that the CP sample exhibited the highest total abundance of pathogens, with 77% of the genera identified as pathogenic. The HP sample had more than 50% of pathogenic genera, while the remaining samples had proportions below 40%. The top 5 pathogenic genera were *Staphylococcus*, *Enterobacter*, *Pseudomonas*, *Klebsiella*, and *Serratia* (Figure 2B). Notably, the abundance of *Staphylococcus* in the CP sample accounted for 98.7% of the total abundance of the top 50 pathogenic bacteria, which was significantly higher than in the other samples. *Enterobacter* and *Klebsiella* exhibited higher abundance in the UC sample, whereas *Enterobacter* and *Pseudomonas* showed higher abundance in the SC sample. Consequently, these results implied that the species composition of the housefly body surface is influenced by environmental factors rather than being inherent to the housefly itself.

### ARG diversity in different housefly body‐surface samples

**FIGURE 3:**
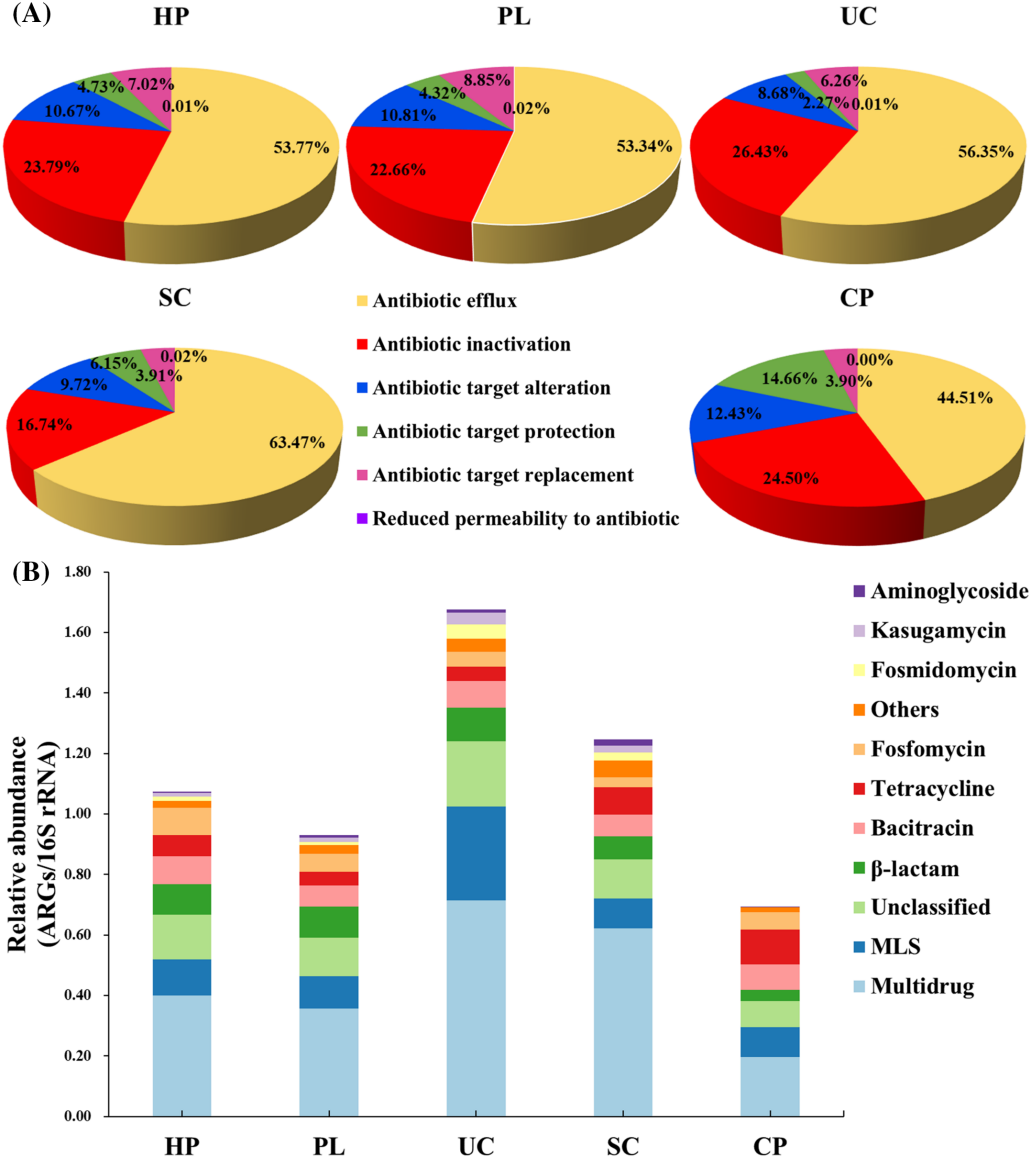
The ARGs profiles in the housefly body‐surface samples. (A) Composition of antibiotic mechanisms for each sample. (B) The top 10 ARG types in each sample.

A total of 420 ARG subtypes belonging to 19 types of ARGs were identified from the 5 housefly body‐surface samples using ARGs‐OAP 2.0 based on clean short reads (Table S4, S5). The ARG types identified covered five major resistance mechanisms, with about 60% of ARGs utilizing antibiotic efflux mechanisms and about 20% utilizing antibiotic inactivation mechanisms, compared to a smaller proportion of ARGs achieving antibiotic resistance through target modification, target substitution, and permeability reduction (Figure 3A).

The abundance of ARGs in the samples ranged from 0.693 to 1.677 ARG copies/16S rRNA gene copies. The ARGs identified in the samples were classified according to antibiotic categories, as shown in Figure 3B. The top 5 predominant ARG types were multidrug resistance (2.29 ARG copies/16S rRNA gene copies), macrolide‐lincosamide‐streptogramin (MLS) resistance (0.734 ARG copies/16S rRNA gene copies), *β*‐lactam resistance (0.429 ARG copies/16S rRNA gene copies), bacitracin resistance (0.407 ARG copies/16S rRNA gene copies), and tetracycline resistance (0.369 ARG copies/16S rRNA gene copies). In addition, the ARG subtypes with higher abundance included *bacA*, *ArlR*, *lnuA*, *crp*, *mecA*, *fosB*, and *mphC*, which accounted for 1.59%–14.21% of the total abundance of common ARGs in each sample. ARGs related to multidrug resistance consistently dominated over other ARGs in these samples.

In total, 738, 909, 895, 1376, and 105 ORFs were annotated as ARG‐like ORFs and carried by 675, 845, 771, 1260, and 97 ACCs in the HP, PL, UC, SC, and CP samples, respectively (Table S2). The abundance of ARG‐like ORFs was calculated using Equation (2). The total ARG abundance on the housefly body surface ranged from 620.4 to 55,898.1 copies/Gb. The identified top 5 abundant types slightly differed from the ARG annotation based on metagenomic analysis directly using clean reads. The heatmap of ARG‐like ORF abundance (Figure S6) demonstrated significant variations in the coverage abundance of each ARG type (Table 1). These results indicated variations in both the abundance and types of antibiotic resistance among the different housefly body‐surface samples.

**TABLE 1:** The abundance of ORFs carrying each type of ARG in the housefly body‐surface samples, normalized by ORF length.

Type	HP	PL	UC	SC	CP
Aminoglycoside	82.93	514.09	1091.359	1754.10	40.13
Bacitracin	1528.49	2144.26	1348.48	4051.93	73.20
Beta‐lactam	1036.66	875.70	2327.54	3084.71	56.75
Bleomycin	0	45.32	74.44	0	0
Chloramphenicol	121.41	172.98	915.41	435.46	13.18
Fosfomycin	428.92	841.58	2162.28	1992.66	57.51
Fosmidomycin	882.67	1710.15	1800.18	1625.38	7.21
Kasugamycin	421.98	1028.13	816.14	2069.58	0
MLS	1555.09	3581.36	5276.35	5025.71	177.74
Multidrug	13,505.37	22,130.32	28,657.96	50,011.84	103.26
Polymyxin	247.63	194.63	1177.15	882.80	0
Quinolone	142.04	65.02	45.70	197.62	0
Rifamycin	0	0	137.76	182.21	0
Sulfonamide	0	47.48	106.69	319.64	2.66
Tetracycline	348.66	277.84	606.04	1155.78	45.96
Trimethoprim	0	0	62.59	89.21	0
Unclassified	1780.62	5883.01	8032.36	6702.83	28.26
Vancomycin	46.08	0	1259.66	1690.49	14.57

### Impact of environmental ARGs on ARGs of housefly body‐surface

**FIGURE 4:**
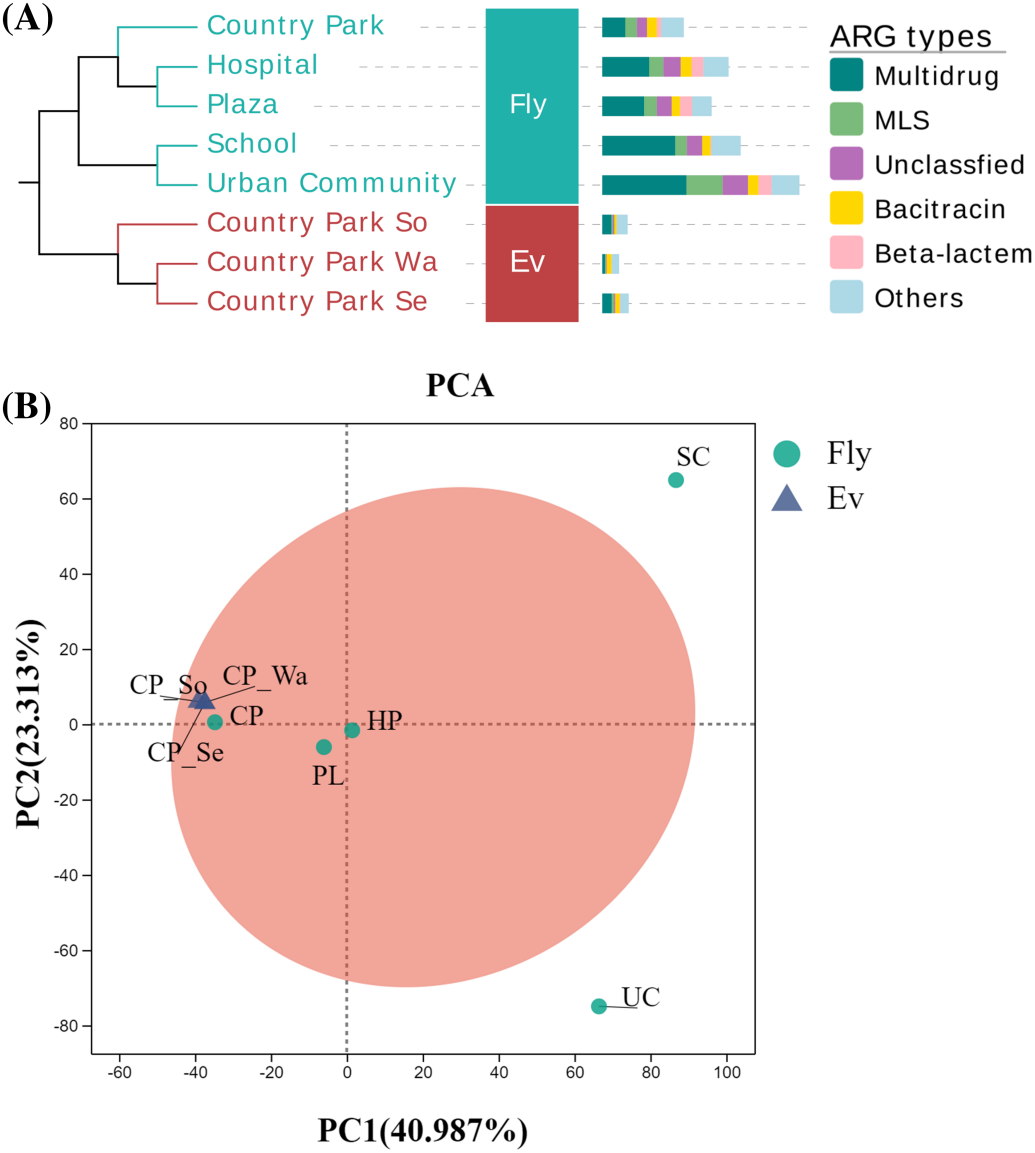
Correlation of ARGs between the housefly body‐surface samples and environmental samples at Country_Park. (A) Hierarchical clustering plots for both sets of samples. The bars show the composition of ARG types for the top 5 abundance for each sample. (B) The PCA score plots of two groups. (Ev: environmental samples; Fly: housefly body‐surface samples).

To further investigate the correlation between the composition of ARGs on the housefly body surface and environmental ARGs, ARG abundances of environmental samples (water, soil, and sediment) from the CP site and all housefly body‐surface samples were clustered. The results of the analysis showed a clear separation between the Fly and Ev groups, with CP samples clustering within the first branch of the separation (Figure 4A). Among the 21 ARG types detected, the type of multidrug resistance, MLS resistance, and bacitracin resistance had a higher percentage of ARGs in both housefly body surface and environmental samples. The changes in the major ARG types corresponded more in both samples. ARG types such as tetracycline resistance, fosfomycin resistance, and β‐lactam resistance, which have lower abundance in environmental samples, can be detected in high abundance in housefly body surface samples. This result did not indicate whether the environment influenced the composition of housefly body‐surface ARGs at the CP site. Therefore, a PCA analysis was then performed on the three environmental samples from the CP site and all housefly body‐surface samples. The results showed that the confidence intervals for the CP environmental samples were nested within the confidence intervals for the housefly body‐surface samples (Figure 4B). Notably, houseflies from the CP (Fly) clustered closer to the group of environmental samples (Ev). This proximity suggests that environmental ARGs influence the composition of ARGs on housefly body surfaces. Furthermore, the environmental ARGs might be enriched on the housefly body surface. Consequently, the results indicate that the housefly body surface might serve as a reflective indicator of environmental ARG distribution, providing a potential means for monitoring ARGs across different geographical areas.

### Health risks of ARGs in the environments

**FIGURE 5:**
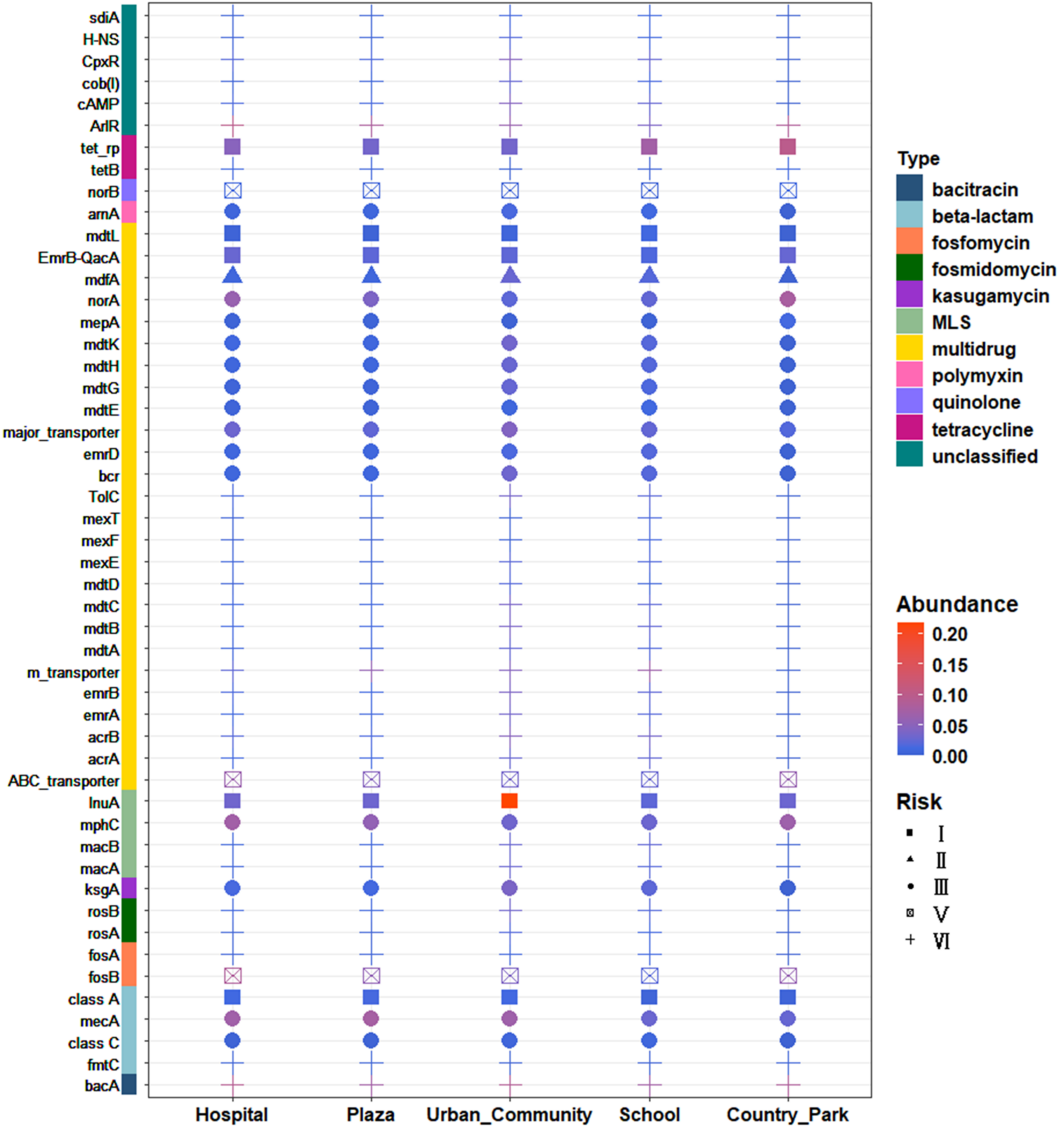
Heatmap of top 50 ARG subtypes and Risk rank in the housefly body‐surface samples collected from five sites. Different colours on the leftmost side indicate different types of ARGs, different shapes indicate different risk ranks of ARGs, and shades of colours indicate high or low abundance.

In our study, a total of 420 ARG subtypes were detected in the housefly body‐surface samples, which were categorized into five levels of risk: Rank I, Rank II, Rank III, Rank IV, and unassessed. Among these ARG subtypes, 29 were identified as high‐risk Rank I ARG subtypes, including *bacA*, *aadE*, *ermB*, *mdtL*, and *tetM*, while 9 were classified as Rank II, including *vatE*, *tetO*, and *penA* (Table S7). These high‐risk ARG subtypes accounted for 1.46%–0.98% of the total abundance. To further assess the risk profile of ARGs in each sampling environment, we analyzed the contribution and abundance of ARGs in each risk class (Figure S7). It was observed that the majority of ARGs in all samples belonged to Rank IV, representing the highest proportion. Rank III ARGs were the second most prevalent in the samples, except for the UC sample. Regarding abundance, when considering the risk of the top 50 ARGs with the highest content in the samples. Among them, 5 were classified as Rank I, belonging to the *β*‐lactam, multidrug, MLS, and tetracycline classes, with mechanisms involving antibiotic efflux and antibiotic inhibition. Additionally, *mdfA* was classified as Rank II, belonging to the multidrug class (Figure 5).

A total of 102 clinically relevant ARG subtypes were identified in all samples (Table S6). These ARGs accounted for 2.93%–8.05% of the total relative abundance of ARGs in the samples. Among the *β*‐lactams, the OXA family of genes responsible for encoding carbapenemases exhibited copy numbers ranging from 0.00014 to 0.00561 (ARG copies/16S sRNA gene copies). Additionally, other β‐lactamase family genes such as *bla*_CTX‐M_, *bla*_SHV_, and *bla*_TEM_ were also detected, although at relatively low levels. Notably, the antibiotic resistance gene (ARGs) *mecA*, typically associated with last‐line treatment, was found to be present in all samples. Interestingly, the PL site, which is located closest to the hospital, exhibited the highest percentage of clinically relevant ARGs detected in the sample. These results suggested that housefly body surfaces can reflect risk profiles in different environments.

### Identification of species carrying ARGs in the environments

The taxonomic annotation results for antibiotic resistance contigs (ARCs), along with the type of ARG carried by each ARC and their abundance, were presented in Table S8. By utilizing BLASTP with the NR database and employing MEGAN annotation, approximately 40.36%– 54.64% of the ARCs across all samples were successfully annotated at the genus level. However, a smaller percentage, specifically 3.09%–9.86%, could be identified at the species level (Table S2).

Species from four different phyla and 32 genera have been identified as hosts for a total of 113 subtypes of ARGs. At the genus level, the top five genera serving as hosts for ARGs are *Enterobacter*, *Klebsiella*, *Staphylococcus*, *Acinetobacter*, and *Pseudomonas*, collectively containing 88 subtypes of ARGs. Notably, *Enterobacter* and *Klebsiella* stand out as the species carrying the most type of ARG subtypes, indicating their significance as reservoirs of antibiotic resistance on the housefly body surface. When comparing these results to the phylum annotation results obtained by using clean reads against the Kraken2 database (Figure 1), no ARGs were detected in *Ascomycota* and *Uroviricota*. However, among the identified species, *Staphylococcus*, *Enterobacter cloacae*, *Klebsiella pneumoniae*, *Enterococcus faecalis*, *E. coli*, and *Acinetobacter baumannii* have been confirmed as human pathogens. These species were consistently found in all samples, except for the CP sample, and were identified as host organisms for ARGs in each sample, with the highest number of identifiable ARG species in the samples collected from the SC site. Moreover, two contigs belonging to *Enterobacteriaceae* in the UC sample carried the most diverse subtypes of ARGs. This observation further highlights the potential of *Enterobacteriaceae* as a reservoir of ARGs on the housefly body surface.

### Co‐occurrence between pathogen and ARGs in the environments

**FIGURE 6:**
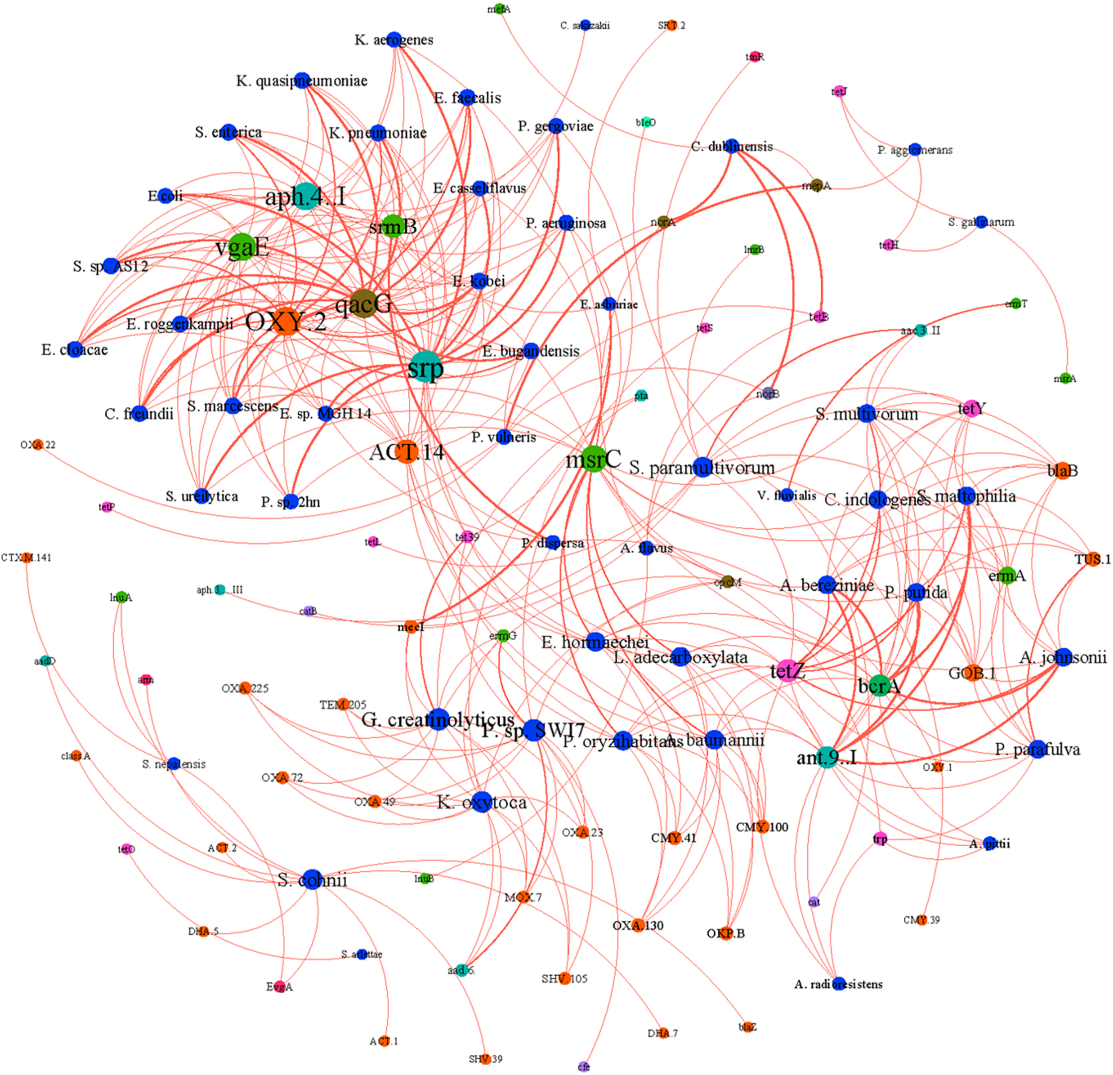
ARG‐ARB network relationship, *r* >0.8 (16S rRNA/copies). Blue: Species, Colour: ARG subtypes, The spot size indicates the level of abundance.

Next, an analysis was conducted on the top 50 pathogenic bacteria present in the total sample, along with all subtypes of ARGs in the sample, to examine their correlation. To visualize the results, network relationship plots were generated (Figure 6). The network relationship plots revealed a positive correlation between multiple subtypes of ARGs in the sample and various pathogenic bacteria. For instance, the streptomycin resistance protein gene of the aminoglycosides class was associated with 7 pathogenic bacteria. Additionally, the multidrug class gene *qacG* was linked to 12 pathogenic bacteria. These results suggest that the housefly body surface can monitor the presence of pathogenic bacteria carrying ARGs in the environment.

### Transmission risk of ARGs in the environments

This study annotated 180 ARCs associated with MGEs, which accounted for 2.25%–23.71% of the total number of ARCs. Among these ARCs, 334 ORFs showed association with ARGs. We identified that 39 ARCs associated with MGEs carried two or more subtypes of ARGs (Table 2). Notably, the CP sample showed the highest correlation with MGEs. To gain insight into the distribution patterns of the identified ARGs in the housefly body‐surface samples, we performed a Plasflow analysis. The analysis showed that 23.82%–37.5% of ARCs (>1000 bp) were located on plasmids, while 32.81%–45.25% of the ARCs were located on chromosomes (Table S2). In this study, a higher number of ARGs associated with aminoglycosides, chloramphenicol, phosphomycin, MLS, quinolones, and tetracycline resistance were detected on the plasmid. In contrast, ARGs associated with multidrug, bacitracin, fosmidomycin, kasugamycin, and polymyxin resistance were predominantly found on chromosomes, suggesting a lower likelihood of their transferability (Figure S8). These findings regarding MGEs suggest that the body surface of houseflies can serve as an indicator of the risk profile for the transmission of ARGs in the environment.

**TABLE 2:** Number of MGE‐related ARCs and ORFs in the housefly body‐surface samples.

	HP	PL	UC	SC	CP
Number of ARCs containing MGE	29	19	66	43	23
Number of ORFs containing MGE	48	29	149	76	32
Number of ARCs containing MGE and more ARG subtypes	4	5	20	6	4

### Impact of population mobility on ARGs/ARBs

The five sampling sites were categorized into three groups based on their population density and level of mobility: the HP site and the PL site with high mobility and medium population density, the UC site and the SC site with low mobility and high population density, and the CP site with medium mobility and low population density. The aforementioned results indicate that in terms of the ARG abundance and diversity, the UC sample and the SC sample have the highest level, while the CP sample has a lower abundance.

## DISCUSSION

Due to the seriousness of antibiotic resistance transmission, it is necessary to monitor the risk of antibiotic resistance in the environment to protect human health. Houseflies are ubiquitous in human life (Iqbal et al., 2014), and their surface microbial communities are affected by habitat (Park et al., 2019). It is attractive to explore whether the housefly body surface can be used as a convenient and comprehensive indicator for environmental risk monitoring. In this study, we conducted a metagenomic analysis of housefly body‐surface samples obtained from five different sites associated with human activity. The correlation between housefly body‐surface samples and typical environmental samples in the risk of ARGs was explored. The species composition of each sample was found to be diverse, with variations in the abundance of different species across different environments (Figure 1). Furthermore, the analysis of ARGs revealed variations in both the abundance and types of ARGs among the sampled sites (Figure 2). This implied that the species and ARGs composition of the housefly body surface were less influenced by intrinsic than external factors. When comparing the ARGs on the housefly body surface with environmental ARGs from the same site, we observed an intrinsic relationship through cluster analysis and PCA analysis (Figure 4). In previous studies, variations in types and quantities of pathogens carried by houseflies collected from different sites were observed (AA et al., 2017; Odetoyin et al., 2020). This observation revealed that the composition of microorganisms and ARGs on the housefly body surface was influenced by the environment, with noticeable differences between samples.

To further demonstrate the comprehensiveness of houseflies for environmental risk monitoring, we utilized them to investigate the risk of ARGs, levels of antibiotic‐resistant pathogens, and the transmission risk of ARGs in the environment. The results regarding ARG risk revealed that the abundance of high‐risk subtypes of ARGs was highest at the UC site (Figure S7). Notably, the most predominant high‐risk subtype was *lnuA*, which has received limited attention in previous studies and is uncommonly chosen in ARG surveillance using qPCR experiments (Song et al., 2021; Zhang et al., 2023). The identification of significant amounts of *lnuA* on the body surface of houseflies in residential areas emphasizes the importance of considering alternative surveillance targets, such as the houseflies, which may provide valuable insights into the presence and potential spread of ARGs in specific environments. Moreover, when identifying species carrying ARGs, a number of pathogenic bacteria at the genus level were detected in all sites (Table S8). *Enterococci* exhibited the highest detection rate, consistent with previous research on pathogenic bacteria found on the housefly body surface (Zurek & Ghosh, 2014). The network analysis revealed positive correlations between multiple subtypes of ARG subtypes and pathogenic bacteria in the environments (Figure 6). Our results are in accordance with other reports that highlight the importance of houseflies in carrying various antibiotic‐resistance pathogenic bacteria (Davari et al., 2010). ARGs have higher mobility if they are found in the same sequence as MGEs. Metagenomic results revealed a strong correlation between ARCs and MGEs in the environments, particularly in the CP site (Table 2). On the surface of houseflies, a coexistence of multidrug, aminoglycoside, *β*‐lactam, and other resistance genes with MGEs were observed, which aligns with the findings from other samples analyzed for MGEs (Yang et al., 2021; Zhao et al., 2021). This suggests that houseflies, as possible environmental indicators, are also capable of detecting the risk of resistance transmission.

Insects, rodents, and pets are reservoirs, vectors, and sentinels of antimicrobial resistance (Gwenzi et al., 2021). A review emphasized the potential of honeybees as AMR sentinels (Resci & Cilia, 2023). However, due to their larger movement radius of 1.5 km compared to the 328–1640 feet of houseflies and their susceptibility to hives, honeybees may not precisely reflect the environmental risk profile of their location. In terms of the average abundance of resistance genes carried by the samples (Table S9), the resistome abundance on housefly body surfaces (1.124 ARG Copies/16S rRNA gene copies) was comparable to livestock wastewater (1.667 ARG Copies/16S rRNA gene copies) and significantly exceeded influent/effluent from wastewater treatment plants (0.452 ARG Copies/16S rRNA gene copies) and fishpond sediments (0.137 ARG Copies/16S rRNA gene copies). Certain resistance gene types (aminoglycoside, MLS, tetracycline) were more prevalent in houseflies versus livestock and sewage samples. The fishpond sediments and wastewater exhibit a high abundance of ARGs due to anthropogenic activity. In contrast, houseflies primarily rely on the surrounding environment for their ARGs. Remarkably, the abundance of ARGs on the housefly body surface surpassed that of them. Furthermore, in terms of ARG abundance at the CP site, the housefly body‐surface samples showed significantly higher levels compared to the environmental samples at the same site. A previous study collected samples of houseflies along with dust, wastewater, and soil in the detection of antibiotic resistance risk in the hen production chain (Zhu et al., 2023). In that study, the highest abundance of ARGs was carried by faeces and houseflies, which provides further evidence that ARGs are more likely to be enriched on the body surface of houseflies. These findings suggest that houseflies amplify the presence of ARGs in the environment and may be better suited as a repository for environmental ARGs. Additionally, the collection of housefly samples is more convenient and straightforward when compared to the sampling method used for environmental samples. Given the diversity of species and ARGs on the housefly body surface, as well as their consistency with the surrounding environment, the housefly body surface has sufficient potential as an environmental risk indicator, and could be considered for future monitoring tools.

Despite the limitations of this study in terms of sample size and types, our findings still suggest intrinsic associations between the microbial communities and ARGs on the housefly body surface and the environment. To further validate the potential of houseflies as a monitor for environmental ARGs, future studies could be improved by increasing the number of sampling sites and sample size and quantifying ARGs using qPCR technology. Finally, sampling could be conducted in different geographic locations and environmental conditions to assess the generalizability of houseflies as a monitor and provide stronger data support for public health and environmental management.

## CONCLUSION

In this study, we employed a metagenomic approach to evaluate the potential of housefly body‐surface samples as convenient and comprehensive indicators for environmental risk monitoring. The abundance of ARGs on the housefly body surface correlated with environmental samples, and the housefly body surface exhibited a higher enrichment of ARGs compared to environmental samples. Furthermore, the housefly body surface demonstrated the ability to reflect risks associated with antibiotic resistance, including the abundance of antibiotic‐resistant pathogens in the environment and the transmission risk of ARGs. These results highlight the feasibility of utilizing housefly sampling as a novel method for monitoring antibiotic resistance in urban environments. By using the housefly body surface to monitor the environment, we can conveniently gain insight into the prevalence and dynamics of antibiotic resistance, ultimately helping to develop effective strategies to mitigate its impact on the environment and human health.

## AUTHOR CONTRIBUTIONS

**Yuhan Yang:** Conceptualization; data curation; formal analysis; investigation; software; methodology; visualization; writing – original draft; writing – review and editing. **Ping Xu:** Funding acquisition; project administration; resources; supervision; validation; writing – review and editing. **Wei He:** Funding acquisition; resources; investigation. **Fei Tao:** Conceptualization; funding acquisition; investigation; methodology; project administration; resources; software; supervision; validation; writing – original draft; writing – review and editing.

## CONFLICT OF INTEREST STATEMENT

The authors declare no conflicts of interest.

## Supporting information


**Figure S1.** Sampling diagram. Five representative sites were selected within Minhang District, Shanghai (1. Hospital, 2. Plaza, 3. Urban Community, 4. School, 5. Country Park). Houseflies were captured using the trap and transferred to the shake flask with PBS.
**Figure S2.** The rank abundance curve of bacterial genus for each housefly body‐surface sample. The different coloured curves indicate different samples, the values in the horizontal coordinate represent the rank of the species, and the vertical coordinate is the normalized species abundance value at the genus level.
**Figure S3.** The α‐diversity measured for the surface of housefly body‐surface samples. The data were analyzed at the genus level at six indices, with the sample name in the horizontal coordinate and the change with each index in the vertical coordinate.
**Figure S4.** Upset plot for bacterial genus on the surface of housefly. The bar chart at the bottom left represents the number of bacterial genera in each sample, the name of each sample in the middle left, the dot matrix at the bottom left corresponds to the bar chart at the top, the black dot represents the shared genus of the collection lines involved, and the bar chart at the top represents the number of bacterial genus corresponding to each intersection.
**Figure S5.** PCoA plots of bacteria of the housefly body‐surface samples. Bacterial communities between each sample as indicated by PCoA plots, each point corresponds to a sample.
**Figure S6.** Heatmap of coverage of ARG‐like ORFs of the housefly body‐surface samples. The colour intensity within the heatmap indicates the abundance of ARG‐like ORFs, with red signifying high abundance and blue indicating low abundance. The notation “***” highlights instances where the ARG‐like ORFs exceed 5000 copies/Gb, emphasizing the ARG types with a notably high prevalence in the samples.
**Figure S7.** ARG risk abundance composition (16S rRNA/copies). Risk I represents the highest level of risk, whereas Risk IV denotes the lowest level of risk.
**Figure S8.** ARG proportion assessment in plasmid and chromosomal sequence.

**Table S1.** Sample information.
**Table S2.** The information about the sequencing data of all samples.
**Table S3.** The abundance of Top 50 pathogenic genus in housefly body‐surface samples (Normalized by reads).
**Table S4.** The abundance of ARG types in housefly samples (copies/16S rRNA).
**Table S5.** The abundance of ARG subtypes in housefly samples (copies/ 16S rRNA).
**Table S6.** List of clinically relevant ARGs according to Majeed et al. (2021) and Prieto Riquelme et al. (2022).
**Table S7.** Risk of ARG subtypes in housefly surface.
**Table S8.** The number of ARGs carried by different genera and species in each sample was identified by MEGAN.
**Table S9.** Abundance of flies and other environmental samples (Yin et al., 2018).

## Data Availability

All data needed to evaluate the conclusions in the paper are present in the paper and/or the Supplementary Materials. The metagenomic genome sequencing data were deposited at NCBI GenBank with the accession number PRJNA1052376.
