# Supplementary material for: Metagenomic analysis reveals houseflies as indicators for monitoring environmental antibiotic resistance genes
Source: Environ Microbiol Rep. 2024 Nov 19;16(6):e70032. doi: 10.1111/1758-2229.70032 (PMC11576324; doi:10.1111/1758-2229.70032)
Supplement: Supplementary file 1 — Figure S1. Sampling diagram. Five representative sites were selected within Minhang District, Shanghai (1. Hospital, 2. Plaza, 3. Urban Community, 4. School, 5. Country Park). Houseflies were captured using the trap and transferred to the shake flask with PBS. Figure S2. The rank abundance curve of bacterial genus for each housefly body‐surface sample. The different coloured curves indicate different samples, the values in the horizontal coordinate represent the rank of the species, and the vertical coordinate is the normalized species abundance value at the genus level. Figure S3. The α‐diversity measured for the surface of housefly body‐surface samples. The data were analyzed at the genus level at six indices, with the sample name in the horizontal coordinate and the change with each index in the vertical coordinate. Figure S4. Upset plot for bacterial genus on the surface of housefly. The bar chart at the bottom left represents the number of bacterial genera in each sample, the name of each sample in the middle left, the dot matrix at the bottom left corresponds to the bar chart at the top, the black dot represents the shared genus of the collection lines involved, and the bar chart at the top represents the number of bacterial genus corresponding to each intersection. Figure S5. PCoA plots of bacteria of the housefly body‐surface samples. Bacterial communities between each sample as indicated by PCoA plots, each point corresponds to a sample. Figure S6. Heatmap of coverage of ARG‐like ORFs of the housefly body‐surface samples. The colour intensity within the heatmap indicates the abundance of ARG‐like ORFs, with red signifying high abundance and blue indicating low abundance. The notation “***” highlights instances where the ARG‐like ORFs exceed 5000 copies/Gb, emphasizing the ARG types with a notably high prevalence in the samples. Figure S7. ARG risk abundance composition (16S rRNA/copies). Risk I represents the highest level of risk, whereas Risk IV denotes t [file EMI4-16-e70032-s001.docx]

**Supplementary Figures**


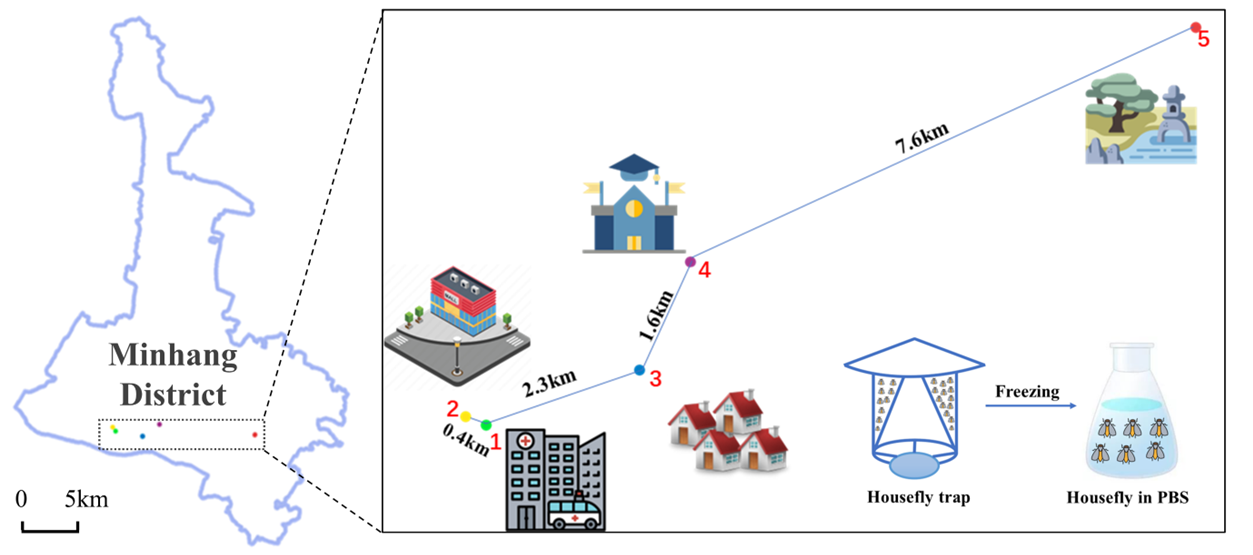


**Fig. S1 Sampling diagram.** Five representative sites were selected within Minhang District, Shanghai (1. Hospital, 2. Plaza, 3. Urban Community, 4. School, 5. Country Park). Houseflies were captured using the trap and transferred to the shake flask with PBS.


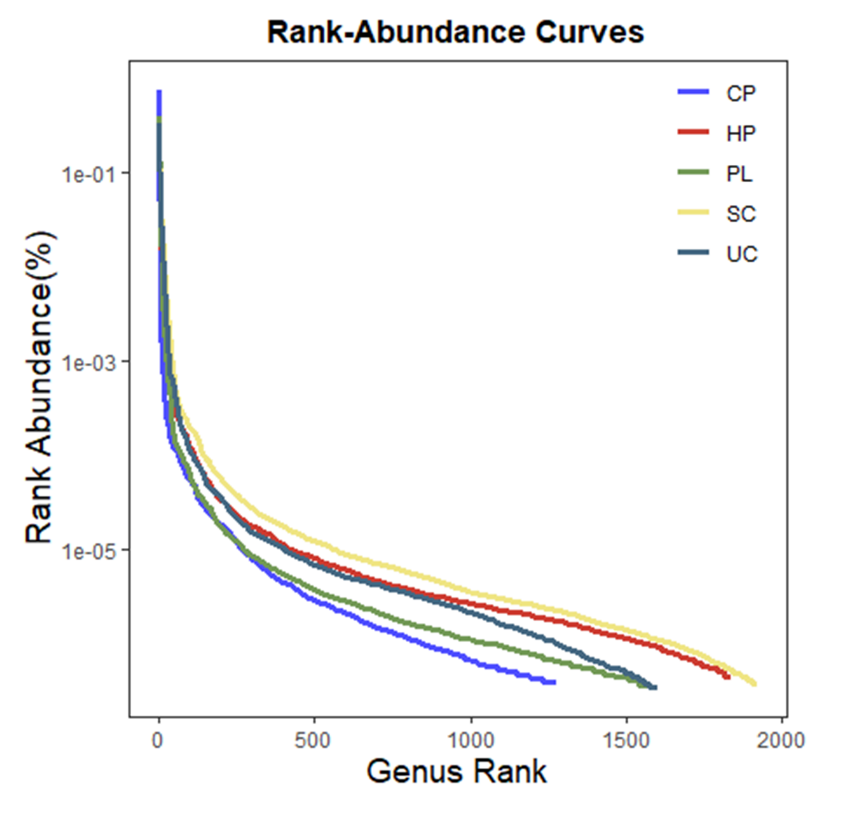


**Fig. S2 The rank abundance curve of bacterial genus for each housefly body-surface sample.** The different coloured curves indicate different samples, the values in the horizontal coordinate represent the rank of the species, and the vertical coordinate is the normalized species abundance value at the genus level.





**Fig. S3 The α-diversity measured for the surface of housefly body-surface samples.** The data were analyzed at the genus level at six indices, with the sample name in the horizontal coordinate and the change with each index in the vertical coordinate.





**Fig. S4 Upset plot for bacterial genus on the surface of housefly.** The bar chart at the bottom left represents the number of bacterial genera in each sample, the name of each sample in the middle left, the dot matrix at the bottom left corresponds to the bar chart at the top, the black dot represents the shared genus of the collection lines involved, and the bar chart at the top represents the number of bacterial genus corresponding to each intersection.


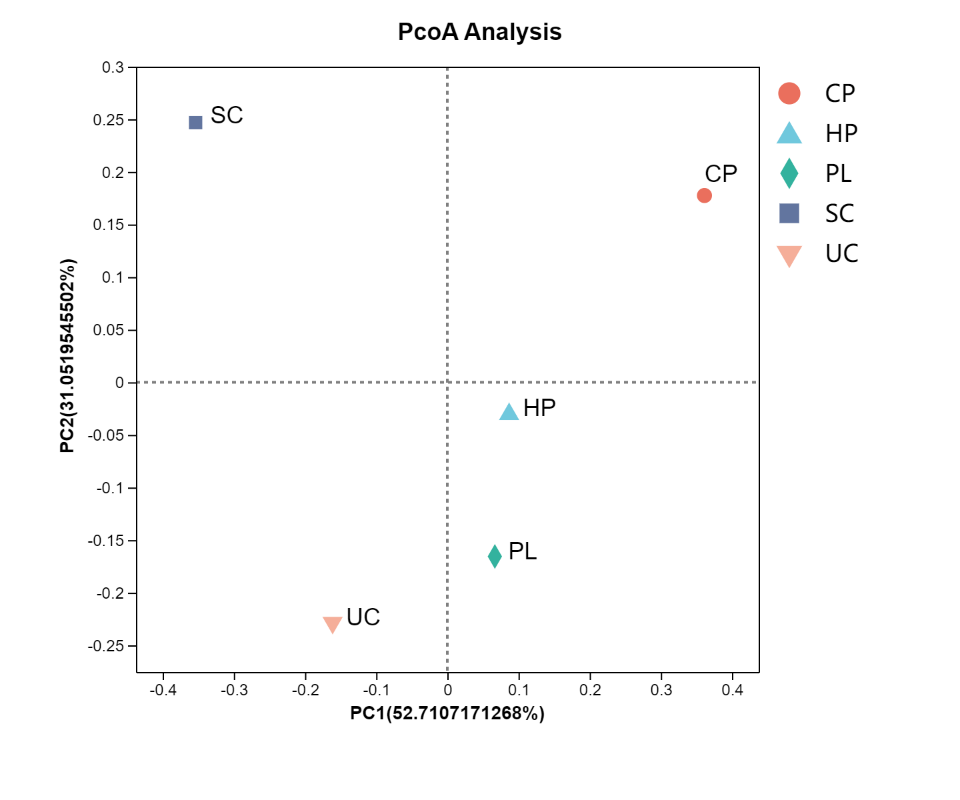


**Fig. S5 PCoA plots of bacteria of the housefly body-surface samples.** Bacterial communities between each sample as indicated by PCoA plots, each point corresponds to a sample.


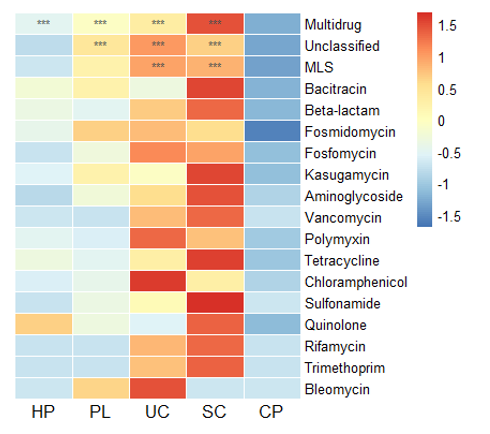


**Fig. S6 Heatmap of coverage of ARG-like ORFs of the housefly body-surface samples.** The colour intensity within the heatmap indicates the abundance of ARG-like ORFs, with red signifying high abundance and blue indicating low abundance. The notation “***” highlights instances where the ARG-like ORFs exceed 5,000 copies/Gb, emphasizing the ARG types with a notably high prevalence in the samples.


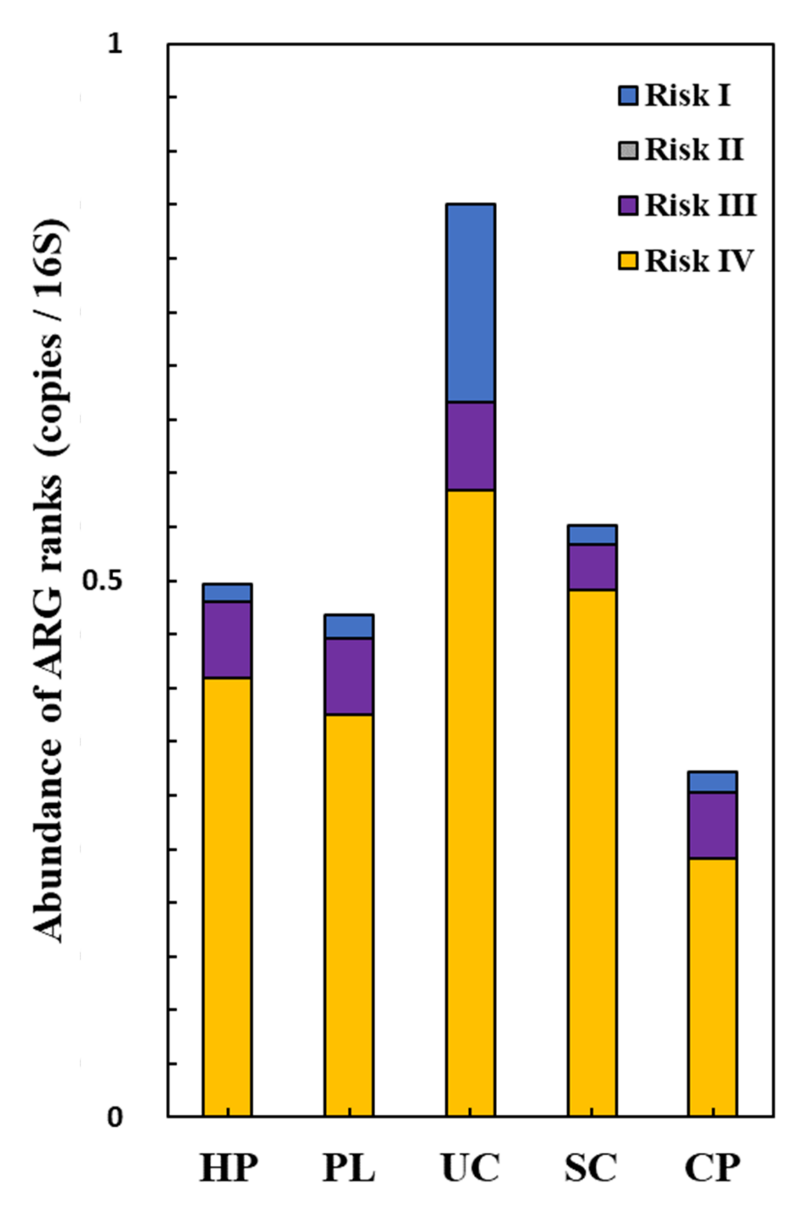


**Fig. S7 ARG risk abundance composition (16S rRNA/copies).** Risk Ⅰ represents the highest level of risk, whereas Risk Ⅳ denotes the lowest level of risk.


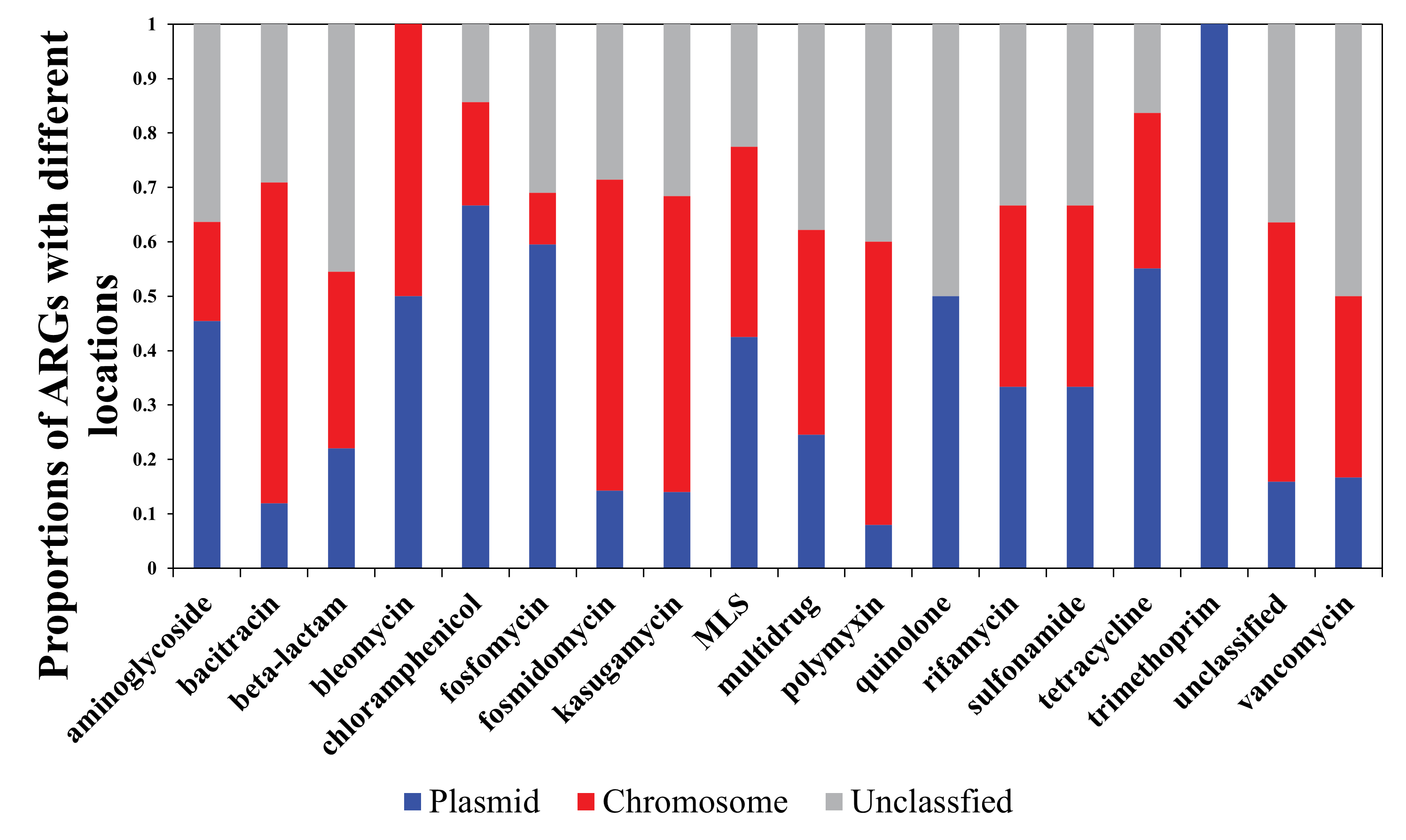


**Fig. S8 ARG proportion assessment in plasmid and chromosomal sequence.**
